# EFFNet: A skin cancer classification model based on feature fusion and random forests

**DOI:** 10.1371/journal.pone.0293266

**Published:** 2023-10-23

**Authors:** Xiaopu Ma, Jiangdan Shan, Fei Ning, Wentao Li, He Li

**Affiliations:** 1 School of Computer Science and Technology, Nanyang Normal University, Nanyang, Henan, China; 2 School of Life Sciences and Agricultural Engineering, Nanyang Normal University, Nanyang, Henan, China; Shanghai Maritime University, CHINA

## Abstract

Computer-aided diagnosis techniques based on deep learning in skin cancer classification have disadvantages such as unbalanced datasets, redundant information in the extracted features and ignored interactions of partial features among different convolutional layers. In order to overcome these disadvantages, we propose a skin cancer classification model named EFFNet, which is based on feature fusion and random forests. Firstly, the model preprocesses the HAM10000 dataset to make each category of training set images balanced by image enhancement technology. Then, the pre-training weights of the EfficientNetV2 model on the ImageNet dataset are fine-tuned on the HAM10000 skin cancer dataset. After that, an improved hierarchical bilinear pooling is introduced to capture the interactions of some features between the layers and enhance the expressive ability of features. Finally, the fused features are passed into the random forests for classification prediction. The experimental results show that the accuracy, recall, precision and F1-score of the model reach 94.96%, 93.74%, 93.16% and 93.24% respectively. Compared with other models, the accuracy rate is improved to some extent and the highest accuracy rate can be increased by about 10%.

## Introduction

Skin cancer is the most common type of cancer, which can be broadly classified into cancers deriving from melanocytes (melanoma) and from the epidermally derived cells (non-melanoma skin cancers/keratinocyte carcinoma) [1]. Among them, melanoma is formed by the rapid multiplication of mutated skin cells. Its shape is similar to that of common nevus, but it has a high degree of invasion, poor prognosis and is very easy to transfer. Although melanoma accounts for less than 5% of all skin cancers, it is implicated in approximately 75% of skin cancer deaths [2]. Statistics indicate that the five-year survival rate of patients in the advanced stage is only less than 20% [3]. The survival rate of skin cancer is increased by 95% when it is detected early [4]. Therefore, the diagnosis of benign and malignant, early and late stages of melanoma plays an extremely important role in the timely treatment of patients.

In the clinical identification, the diagnosis of melanoma can be performed with the aid of dermatoscopy. Some studies have shown that the use of dermoscopic techniques can help dermatologists improve the diagnostic accuracy by 5% and 30% [5]. Doctors combined the image structure of dermoscopic images to analyze the process of lesions in the skin lesion area, and then summarized a number of melanoma diagnostic rules, such as: the 7-point checklist [6], Menzies method [7], ABCD rule [8], and CASH rule [9]. Despite these criteria, the diagnosis of skin cancer mainly relies on years of experience of doctors, which requires doctors to have clinical experience and a great deal of professional knowledge. Moreover, there are many types of skin cancer with different shapes and frequently changing appearance, which can easily cause errors even for experienced experts facing dermoscopic images of skin lesions with large intra-class disparities and small inter-class disparities. Therefore, the introduction of computer-aided diagnosis (CAD) technology has extremely high practical significance for improving the speed and accuracy of skin cancer recognition.

CAD technology for skin cancer identification is mainly carried out through four steps, namely preprocessing, lesion segmentation, feature extraction and classification. Early CAD technology was mainly based on traditional machine learning methods to identify skin cancer by extracting features such as shape, color, boundary, symmetry, and texture of the lesion area for classification. However, this method is affected by background complexity and contrast noise, which leads to a decrease in the accuracy of identification, and the process of extracting effective features is relatively cumbersome. In recent years, with the development of deep learning, researchers have adopted deep learning-based CAD systems for skin cancer identification. Deep learning can automatically excavate the deep nonlinear relationships in medical images, extract features, and eliminate complex structures in feature engineering steps. With strong adaptability and portability, it is easier to be applied to skin disease recognition [10]. However, in the traditional deep learning methods, there are still some disadvantages such as unbalanced datasets leading to model overfitting, redundant information in the extracted features, and the neglect of some feature interactions between different convolutional layers. Based on this, we propose the EFFNet based on feature fusion and random forests (RF) [11] to overcome these disadvantages.

The main research content and organization of this paper are as follows:

1. We use transfer learning to train the model to overcome the disadvantage of model overfitting. By using the weights trained by the EfficientNetV2 model [12] on the ImageNet dataset [13], fine-tuning is performed on the target dataset to achieve the purpose of reducing model training time and improving model training speed.
2. For the classification model ignoring the interaction relationship between some features between layers, which leads to the insufficient utilization of features, the hierarchical bilinear pooling (HBP) [14] is used to fuse the features. By fusing features of different levels, the interactions of some features between layers can be captured, so as to enhance the expressive ability of features.
3. For the disadvantage of redundant information in the features extracted by the EfficientNetV2 model, we add an efficient channel attention (ECA) [15] mechanism before HBP for feature selection or weighting to reduce the interference of redundancy and noise information.
4. We utilize RF for classification prediction. RF can overcome the disadvantage of imbalance in the HAM10000 dataset [16] and avoid overfitting of the model, thus improving the generalization ability of the model.

The paper is organized as follows. The Related works section summarizes the research work on the classification of skin cancer. The Proposed methods section introduces the model named EFFNet and advantages used in this paper. The experimental dataset, environment configuration and evaluation metrics are introduced in the Experiments section. The Results and discussion section carries out specific experiments on the model proposed in this paper and analyzes the experimental results concretely. Finally, the Conclusion section conducts the model of this paper and future work.

## Related works

Early CAD techniques were mainly based on traditional machine learning methods to classify skin cancer by extracting features such as lesion area shape, color, boundary, symmetry, and texture. Among them, Hameed et al. [17] proposed a multiclass skin lesion classification framework for classifying multiclass and prominent skin lesions. The framework extracts 35 different features from the segmented region of interest (ROI) and finally trains the classification model using different classifiers. Murugan et al. [18] used the watershed method for segmentation, then combined the ABCD rule and the Gray Level Co-occurrence Matrix (GLCM) method for feature extraction, and finally used support vector machine (SVM) and RF for classification. However, the early feature extraction relies on professional knowledge and experience, which is often ineffective for complex dermoscopic images, and is also affected by noise such as contrast, resulting in low efficiency and poor generalization.

In recent years, the ability of deep learning to automatically extract features, which is highly adaptive and portable, has led to its widespread use in medical imaging. Among them, Gajera et al. [19] proposed an automated framework that uses a pre-trained deep convolutional neural network model to extract visual features from dermoscopic images and then uses a set of classifiers to detect melanoma. Maduranga et al. [20] proposed an artificial intelligence-based mobile application for skin disease type detection, using the MobileNet network with migration learning for fast identification. Khan et al. [21] proposed a CAD method based on deep learning, which preprocessed the skin lesions through decorrelation formula technology, and further used Mask Region-based Convolutional Neural Network (MASK-RCNN) for segmentation. After that, the resultant segmented images were passed to the DenseNet deep model for feature extraction. Two different layers, average pool and fully connected, are used for feature extraction, which are later combined, and the resultant vector is forwarded to the feature selection block for down-sampling using proposed entropy-controlled least square SVM (LS-SVM). However, the accuracy of the above models is relatively low on the multiclassification dataset.

To further improve the accuracy of the model for multi-classification, some people have made improvements in feature extraction and feature fusion. Qian et al. [22] proposed a deep convolutional neural network dermoscopic image classification method based on multiscale attention block grouping (GMAB) and class-specific loss weighting to enhance fine-grained features by extracting multiscale static features using GMAB. The method can achieve an accuracy of 91.6% on the HAM10000 dataset, but the proposed model has certain limitations in sensitivity. Xin et al. [23] proposed a skin cancer classification network SkinTrans based on Vision Transformers (VIT), and used multi-scale visual transformation for feature extraction, which achieved an accuracy of 94.3% on the HAM10000 dataset. Afza et al. [24] proposed to select the best features using a hybrid of whale optimization and entropic mutual information (EMI) methods, then fuse the selected features with an improved typical correlation method, and finally use an extreme learning machine based classification. This feature selection method improves the computational efficiency and accuracy, and its accuracy on the HAM10000 dataset is 93.4%. Calderon et al. [25] proposed a bilinear CNN method consisting of ResNet50 and VGG16 architectures and improved the generalization of the model by adapting it to new data through migratory learning and fine-tuning, which eventually achieving 93.21% accuracy on the HAM10000 dataset. Although the above methods improve the accuracy of model multi-classification by improving feature extraction or feature fusion, they ignore the interactions between different convolutional layers and the redundant information in the extracted features. Therefore, we introduce the ECA mechanism and HBP. Feature selection or weighting is carried out by ECA mechanism to reduce the interference of redundant and noisy information. At the same time, HBP is used to carry out feature fusion. HBP can capture the interactions of some features between layers by integrating features of different levels and enhance the expression ability of features, thereby improving the accuracy of model classification.

## Proposed methods

**Fig 1:**
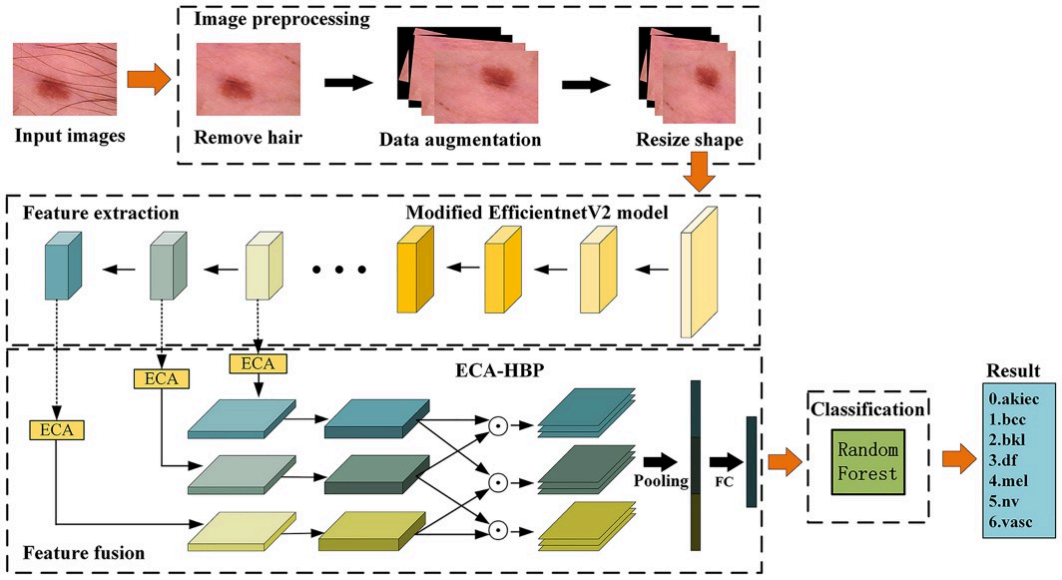
Overall structure of EFFNet.

The overall structure of EFFNet is shown in Fig 1. The model includes four functional modules: image preprocessing, feature extraction, feature fusion and classification. The pseudo code of the model is as follows:

**Algorithm: 1** EFFNet framework

**Require**: Given image *I*, feature extraction model *M*_*e*_, feature fusion *M*_*f*_, classification *M*_*r*_

**Ensure**: R

 1: Convert *I* into grayscale image

 2: Perform blackhat morphological filtering on grayscale image

 3: Use the threshold function for thresholding processing to obtain mask

 4: **if** pixel value < 10 **then**

 5:  set pixel value = 0

 6: **else**

 7:  set pixel value = 255

 8: **end if**

 9: Inpaint the original image *I*′ depending on the mask

 10: Perform data enhancement on image *I*′ and resize to 448 × 448 to obtain *I*″

 11: Pass *I*″ through *M*_*e*_

 12: Get three feature maps *R*_1_, *R*_2_, *R*_3_

 13: Pass *R*_1_, *R*_2_, *R*_3_ through *M*_*f*_

 14: Get feature map of fully connected layer F

 15: Pass F through *M*_*r*_

 16: Get classification results R

 17: **return** R

### Image preprocessing

In view of unbalanced HAM10000 dataset and blurred boundary, irregular shape, low contrast with surrounding skin and hair noise in the lesion area of dermoscopic images, we use hair noise removal, data enhancement and image adjustment to achieve image preprocessing and prevent model overfitting.

**Fig 2:**
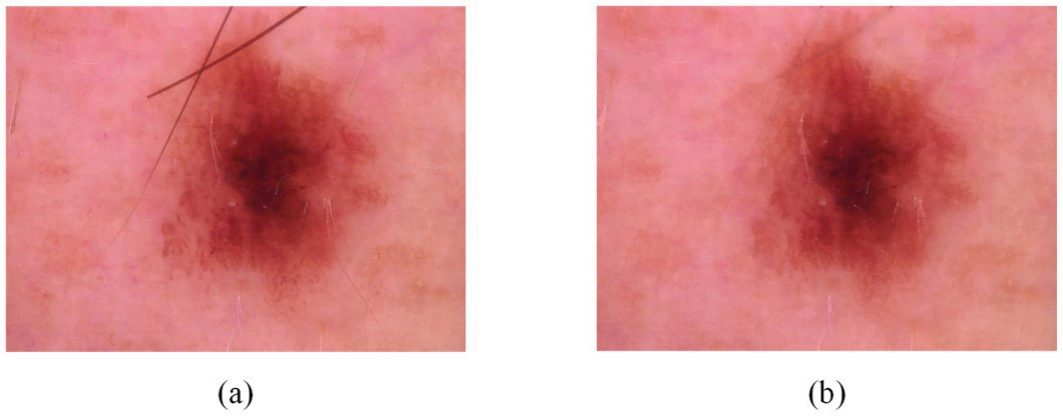
Comparison of hair noise removal. (A)is the original image and (B) is the image after hair noise removal.

**Fig 3:**
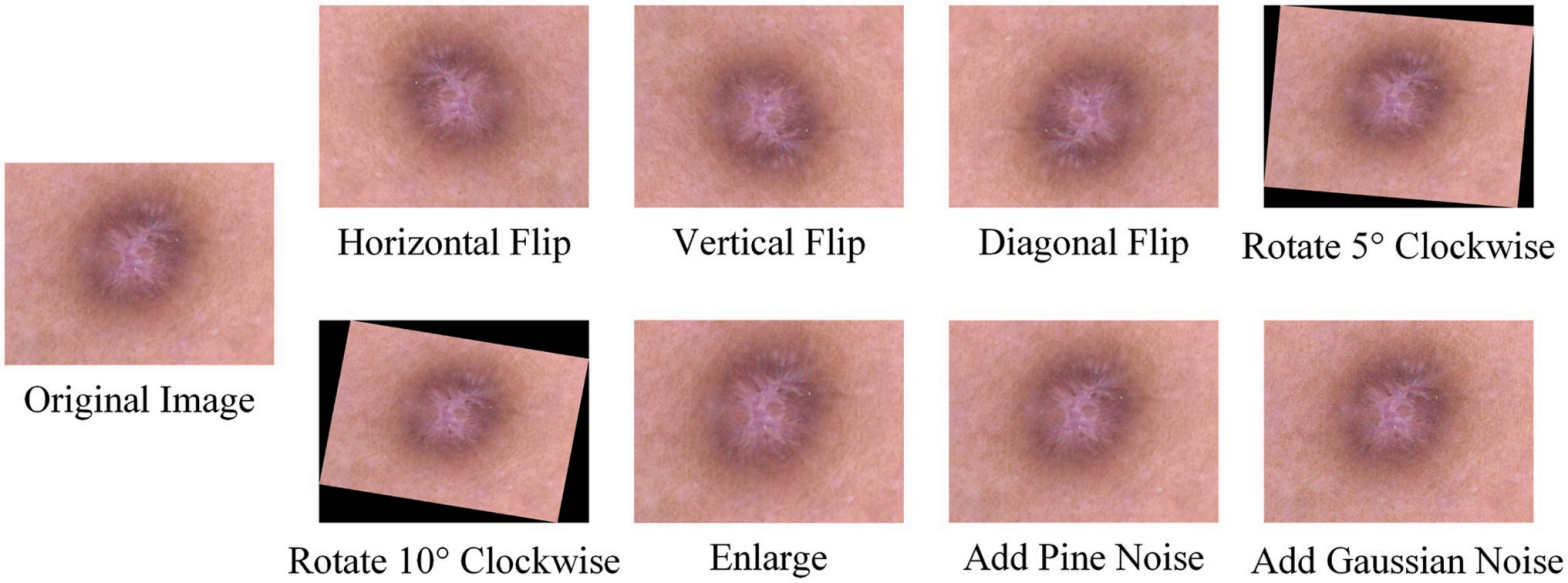
Image enhancement.

**Fig 4:**
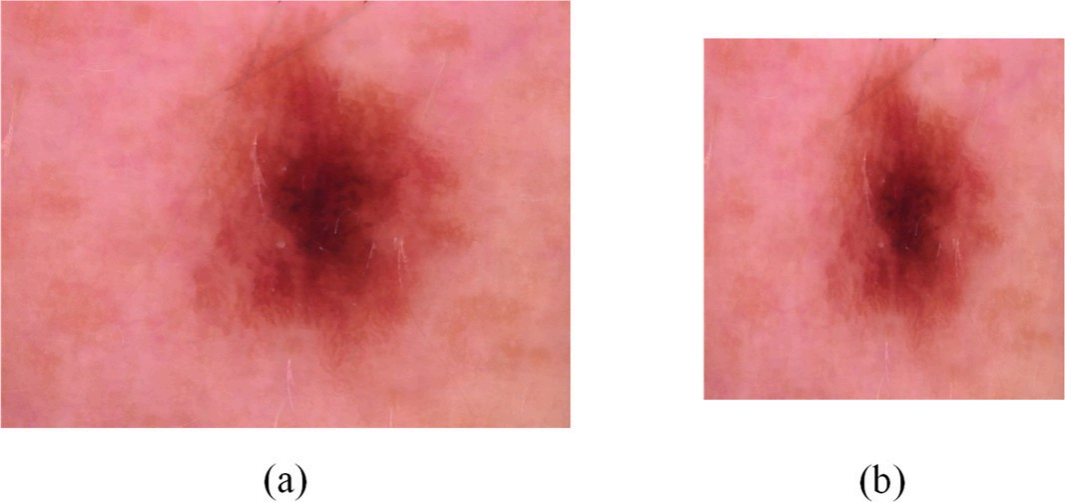
Comparison of cropped images. (A) is the original image and (B) is the cropped image.

1. Hair noise removal: We use the morphological filtering method to remove hair noise from dermoscopic images. Firstly, we convert the dermoscopic image into gray image. Then, the gray image is transformed by the morphological black cap with a structure element of 17 × 17. After that, we create a mask for the inpainting task. Finally, the image inpainting algorithm is applied to inpaint the image. The comparison before and after image removal of hair noise is shown in Fig 2.
2. Data enhancement: We perform horizontal flip, vertical flip, diagonal flip, enlarge, rotation (0°-30° angle), add Gaussian noise, add pine noise and other operations on the training set. Through data enhancement, we can achieve the balance of 7 categories on the HAM10000 dataset, so as to achieve a better classification effect. Fig 3 shows the comparison of images enhanced in different ways.
3. Image adjustment: Finally, we uniformly resize the images to 448×448 to improve model training efficiency. A comparison of the before and after cropping images is shown in Fig 4.

### Feature extraction

The significance of features extracted by different models lies in their ability to capture distinct and valuable information from the input data. Each feature extraction model may emphasize different aspects of the data, leading to a richer and more comprehensive representation. This paper utilizes the EfficientNetV2-M [12] network model for feature extraction, which is based on the EfficientNetV2 network architecture, and offers the following advantages:

1. It employs a balanced approach to network depth and width. This enhances the model’s representational power while avoiding over-parameterization and excessive computation.
2. It employs a compound scaling strategy that optimizes depth, width, and resolution simultaneously. This approach yields strong performance across different tasks and datasets.
3. It achieves impressive performance without excessively increasing model size and computational complexity.

**Fig 5:**
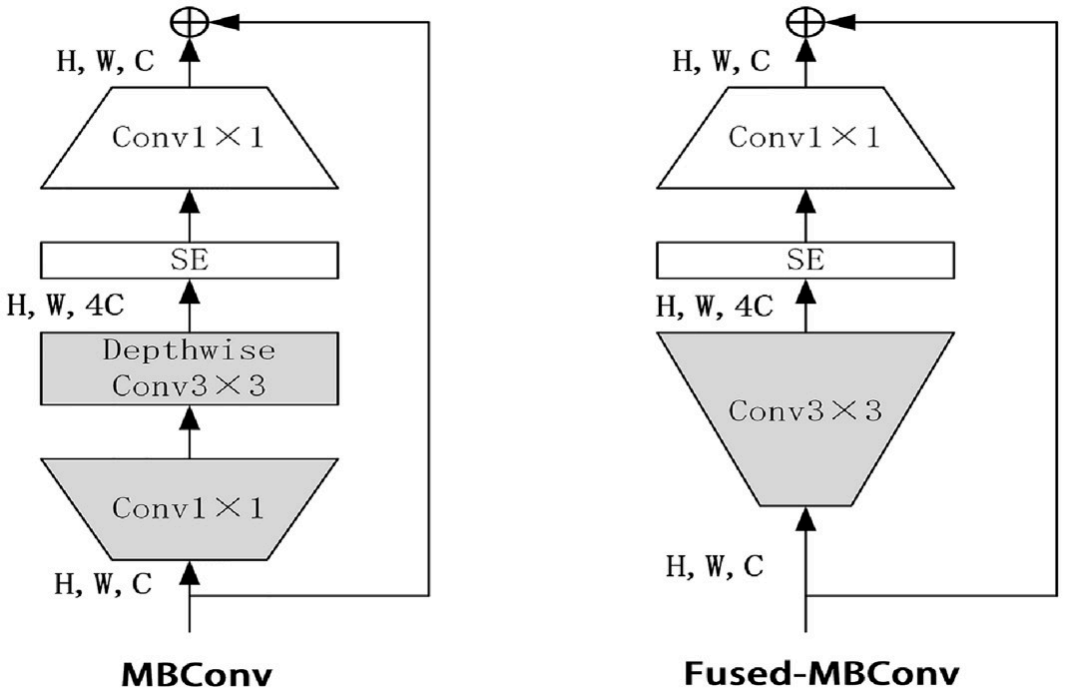
MBConv layer and Fused-MBConv layer architecture.

The EfficientNetV2-M model is mainly composed of a series of MBConv modules and Fused-MBConv modules. The structures of these two modules are shown in Fig 5. Among them, the Fused-MBConv module replaces the Depthwise Conv in the original MBConv module and the upscaled Conv1×1 with Conv3×3. Although Depthwise Conv has fewer parameters and less calculation than ordinary convolution, usually some available accelerators can not be fully utilized. The model test found that using Fused-MBConv module in the shallow network can make the model fully utilize some existing accelerators, thus improving the training speed.

**Fig 6:**
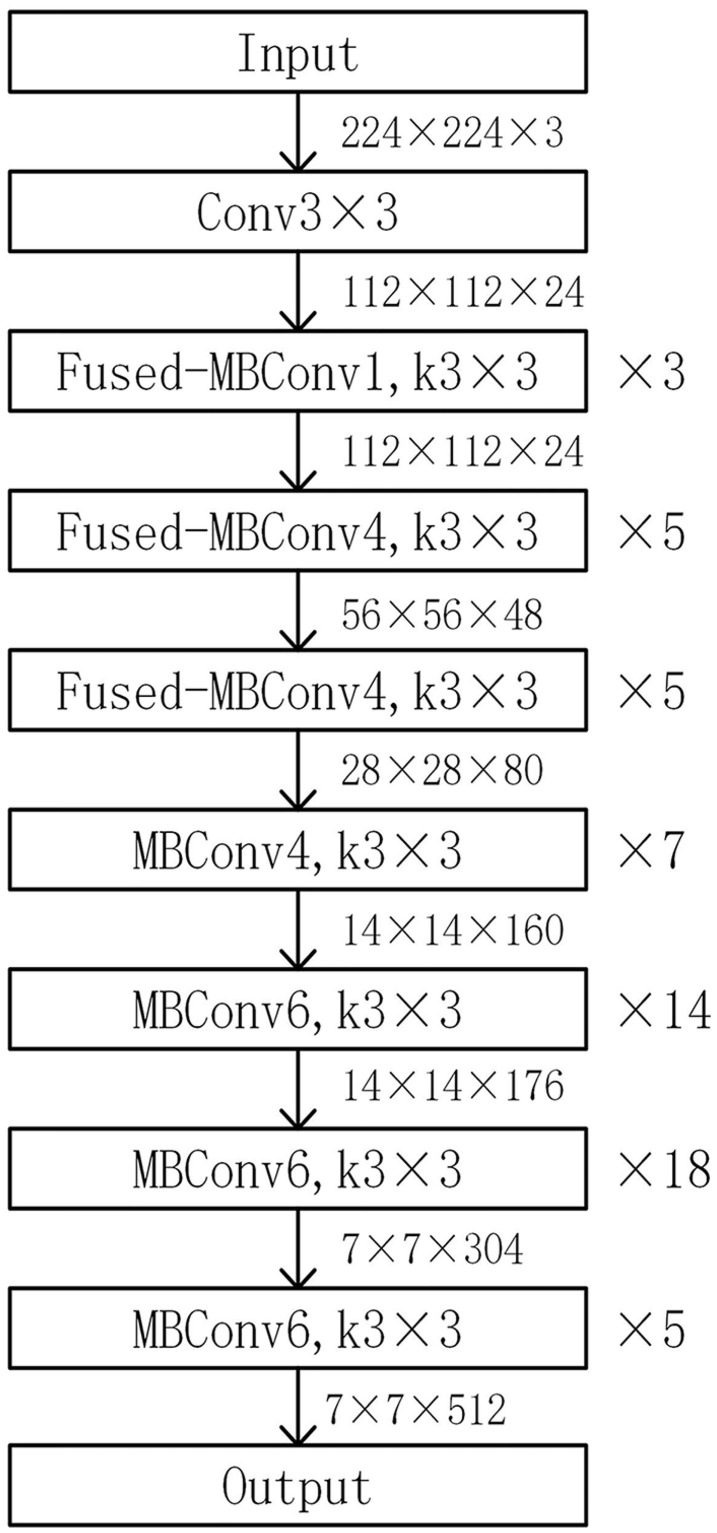
Modified EfficientNetV2-M model structure.

When feature extraction is performed using the EfficientNetV2-M model, the last several layers of the original model are removed and the feature extraction part is retained, thus improving the training speed of the model, reducing the model parameters, preserving the feature extraction ability and facilitating model expansion and fine-tuning without affecting performance. The modified EfficientNetV2-M model is shown in Fig 6.

### Feature fusion

Skin cancer lesion recognition is a fine-grained visual recognition, characterized by small inter-class differences and large intra-class differences. In order to solve the disadvantage of insufficient feature utilization caused by the classification model ignoring part of the feature interactions between layers, we add feature fusion after the modified EfficientNetV2 model. We pass the feature maps obtained from the last three MBConv modules of the EfficientNetV2 model into the feature fusion. By integrating features of different levels to capture the interactive information of some features between layers, the expression ability of features is enhanced, and the accuracy of the model in lesion recognition is further improved.

#### Hierarchical bilinear pooling

**Fig 7:**
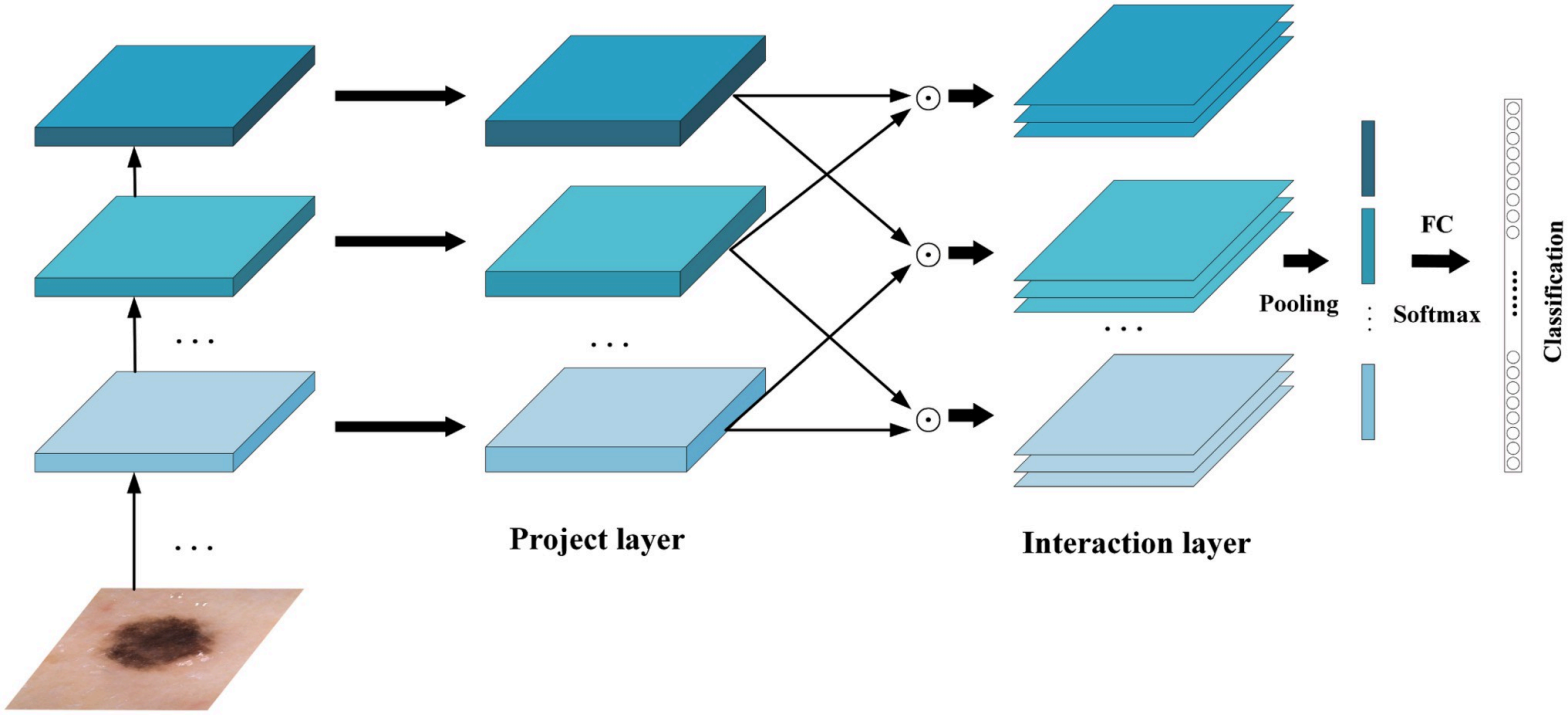
Hierarchical bilinear pooling network architecture.

HBP is a feature fusion method. The core of the method is to incorporate more features of the convolutional layer by cascading multiple cross-layer bilinear pooling. Among them, the cross-layer bilinear pooling is mainly divided into interaction and classification stages, and its formula are Eqs (1) and (2):

$$\begin{array}{c} {\text{z}}_{\text{int}\;}=U^{T}\text{x}\circ V^{T}\text{y} \end{array}$$ (1)

$$\begin{array}{c} \text{z}=P^{T}{\text{z}}_{\text{int}\;}\in R^{o} \end{array}$$ (2)

where $U\in R^{c\times d}$ and $V\in R^{c\times d}$ are projection matrices, $P\in R^{d\times o}$ is the classification matrix, ∘ is the Hadamard product and *d* is a hyperparameter deciding the dimension of joint embeddings. It is found that the inter-layer feature interaction between different convolutional layers is beneficial to capture the discriminative partial attributes between fine-grained subcategories. Therefore, multiple **z**_int_ of the cross-layer bilinear pooling are spliced to obtain the interaction features of the HBP. The final output of the HBP can be derived by Eq (3):

$$\begin{array}{c} {\text{z}}_{\text{HBP}}=HBP\left(\text{x},\text{y},\text{z},\ldots \right)=P^{T}{\text{z}}_{\text{int}\;}=P^{T}concat\left(U^{T}\text{x}\circ V^{T}\text{y},U^{T}\text{x}\circ S^{T}\text{z},V^{T}\text{y}\circ S^{T}\text{z},\ldots \right) \end{array}$$ (3)

where *P* is the classification matrix, *U,V,S*,… are the projection matrices of the convolutional layer feature **x**, **y**, **z**,… respectively. The overall flowchart of the HBP framework is illustrated in Fig 7.

#### Efficient channel attention mechanism

ECA is a lightweight attention mechanism that is often applied in visual models. Compared with traditional attention mechanisms (e.g. Squeeze-and-Excitation(SE) attention mechanism [26]), the ECA mechanism is more efficient and simple, with strong generalization ability and performance improvement. ECA mechanism considers that the dimensionality reduction operations employed in the SE attention mechanism negatively affect the prediction of channel attention, while obtaining the dependencies of all channels is inefficient and unnecessary. Therefore, based on the SE attention module, the ECA mechanism changes the fully connected layer to 1 × 1 convolution to learn channel attention information. By using 1×1 convolution to capture information between different channels, channel dimension reduction can be avoided while learning channel attention information, and the amount of parameters is also reduced.

**Fig 8:**
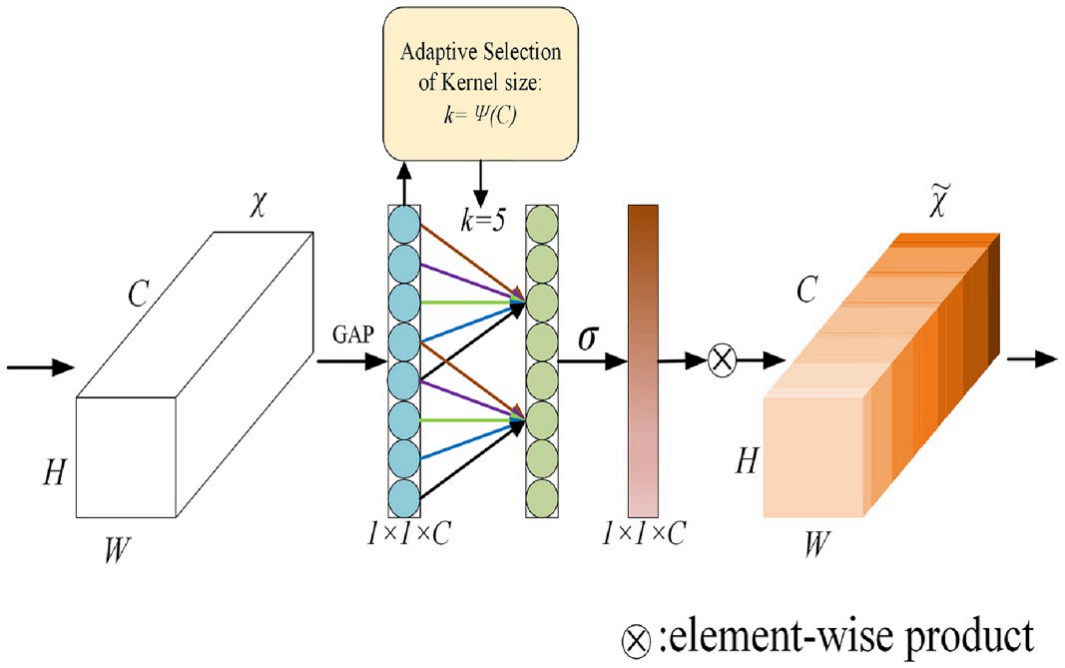
Structure diagram of efficient channel attention mechanism.

Fig 8 shows the structure diagram of ECA mechanism. First, dimension of the input feature map is H × W × C, then the feature map is compressed in the spatial dimension using global average pooling (GAP) to obtain a 1 × 1 × C feature map. After that, the compressed feature map is subjected to channel feature learning by 1 × 1 convolution. Finally, the feature map with channel attention is multiplied channel by channel with the original input feature map to output a feature map with channel attention. Where, when performing convolution operations, the size of its convolution kernel will affect the receptive field. Because the correlation between different channels changes dynamically, it is difficult to adapt to the dynamic changes with fixed convolution kernels. Therefore, when extracting different ranges of features, the ECA mechanism uses dynamic convolution kernels to do 1×1 convolution, so as to learn the importance between different channels, improve the representation ability of features and avoid information loss. The adaptive function of the convolution kernel is defined as Eq (4):

$$\begin{array}{c} k=\psi (C)=|\frac{{\text{log}}_{2}(C)}{\gamma }+\frac{b}{\gamma }{|}_{odd} \end{array}$$ (4)

where *k* denotes the convolutional kernel size, *C* denotes the number of channels, ||_*odd*_ denotes that *k* can only take odd numbers, and *γ* and *b* are set to 2 and 1 in the paper to change the ratio between the number of channels *C* and the convolutional kernel size.

#### Hierarchical bilinear pooling based on efficient channel attention mechanism

HBP can capture the properties of different object parts by mapping input features to high-dimensional space. However, there may be some redundant or useless information in the extracted features before they are mapped to high-dimensional space. Therefore, feature selection or weighting of the data is required to retain features that are more important to the classification task, thereby improving the performance of the model.

**Fig 9:**
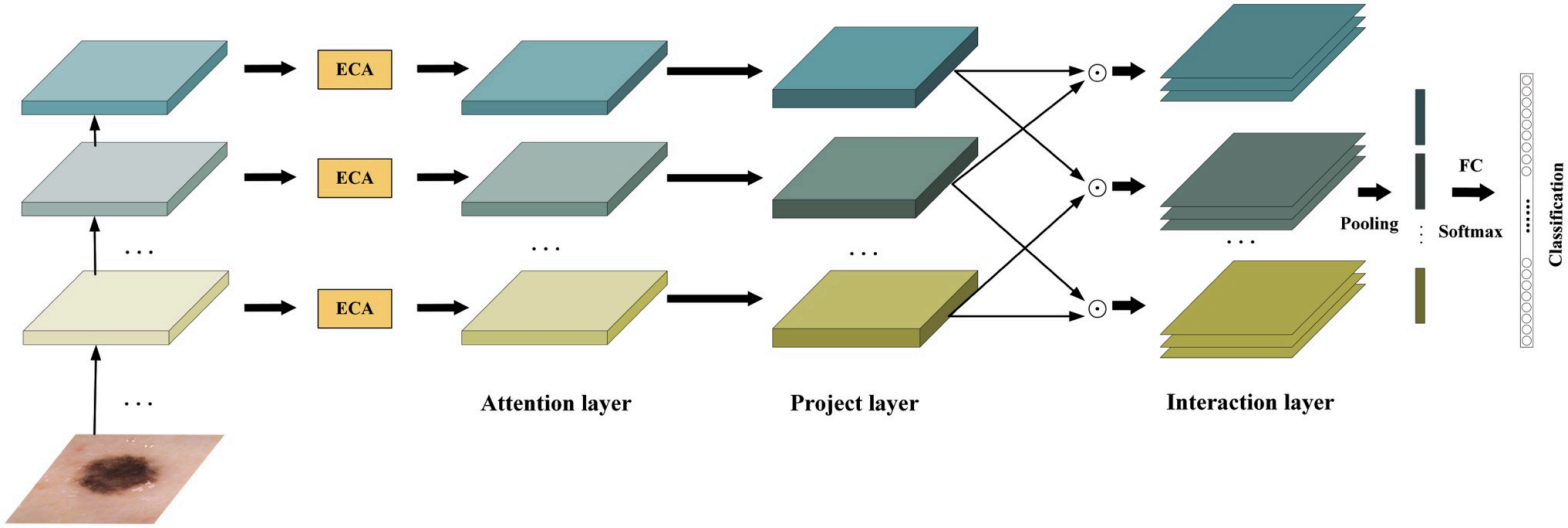
ECA-HBP structure diagram.

The attention mechanism enables the model to pay more attention to the features that are more important to classification task. By adding an attention mechanism to HBP, features of original data can be selected or weighted before mapping the data to high-dimensional space. Specifically, the attention mechanism can achieve this goal by learning a set of weights, which can indicate the importance of each feature in the input data. In this way, the model can reduce the interference of redundant and noisy information, thereby improving the model performance. Different from traditional feature selection or weighting methods, attention mechanism can adaptively adjust the weights to better adapt to different input datas and task requirements. The integration of ECA within HBP can potentially enhance the cross-channel interaction at both the local and hierarchical levels. This means that the model can better capture relationships between channels within individual layers as well as across different layers, resulting in more comprehensive feature representations. In this paper, the improved HBP is named ECA-HBP, and the flow chart of ECA-HBP is shown in Fig 9.

### Classification

**Fig 10:**
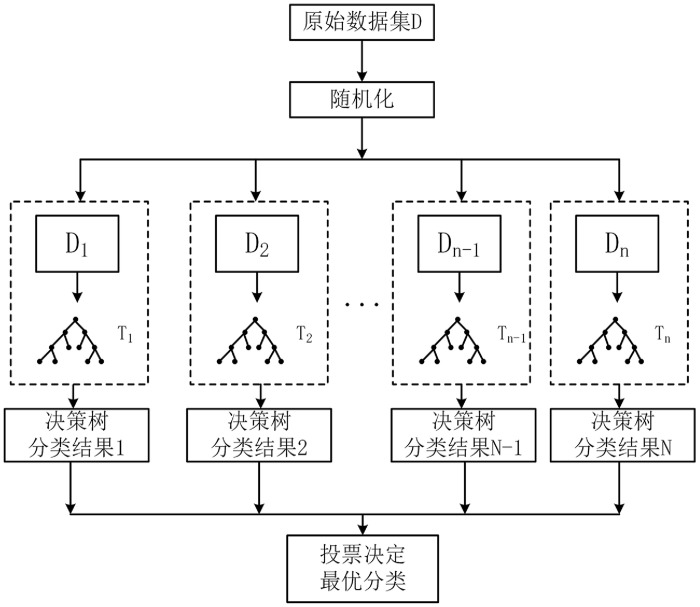
Schematic diagram of random forests algorithm.

RF is a classification model proposed by Breiman in 2001, which consists of a number of decision trees integrated, using multiple trees to train and predict the samples, and the final classification result is decided by a vote of the multi-tree classifier. In this paper, RF algorithm is used to classify the fused features. RF algorithm consists of two steps: the establishment of the RF and the prediction of the RF algorithm. The schematic diagram of the RF algorithm is shown in Fig 10.

RF can train multiple decision trees by randomly selecting data subsets and feature subsets, which can deal with unbalanced datasets and reduce the risk of overfitting, thus improving the generalization ability of the model. In order to find a better combination of super parameters and improve the performance of the model, the super parameters of the RF classifier are adjusted and optimized by using Bayesian optimization method when using RF.

## Experiments

### Experimental dataset

This paper uses the HAM10000 dataset for experiments, which contains 10,015 dermoscopic images of pigmented skin lesions, which can be classified into seven important lesion categories: melanocytic nevus (nv), melanoma (mel), benign keratosis (bkl), basal cell carcinoma (bcc), actinic keratosis (akiec), vascular lesion (vasc), and skin fibroma (df). The dataset includes images collected from different sources and patients, enhancing its representativeness of real-world skin lesions. The HAM10000 dataset is divided into training set and test set according to the ratio of 8: 2. Table 1 shows the distribution of each category in the HAM10000 dataset.

**Table 1:** Distribution of different types of data in the dataset.

Type	Train	Test	Total
*nv*	5364	1341	6705
*mel*	890	223	1113
*bkl*	879	220	1099
*bcc*	411	103	514
*akiec*	261	66	327
*vasc*	113	29	142
*df*	92	23	115
*Total*	8010	2005	10015

### Experimental details

The hardware environment of this experiment is Intel(R) Xeon(R) Platinum 8350C CPU @ 2.60GHz, 42G RAM, NVIDIA GeForce RTX 3090 GPU; the software environment is Ubuntu20.04 system, Python3.8, Cuda 11.3, Pytorch 1.11.0. During the training process, we use the cross-entropy loss function for skin cancer lesion classification task. Initialize the hyperparameters, where the learning rate, epoch, and batch size are 0.001, 100, and 8, respectively. The model uses the SGD optimizer with the momentum of 0.9 and the weight decay of 5e-5.

### Evaluation metrics

The Accuracy (Acc), Precision (Pre), Recall, and F1-score are used to evaluate the classification efficiency of the proposed model.The formulas are Eqs (5), (6), (7) and (8), respectively.

$$\begin{array}{c} \text{Acc}=\frac{TP+TN}{TP+TN+FP+FN} \end{array}$$ (5)

$$\begin{array}{c} \text{Pre}=\frac{TP}{TP+FP} \end{array}$$ (6)

$$\begin{array}{c} \text{Recall}=\frac{TP}{TP+FN} \end{array}$$ (7)

$$\begin{array}{c} F1-\text{score}=\frac{2\times Pre\times Recall}{Pre+Recall} \end{array}$$ (8)

Among the correct sample categories, *TP* (True Positive) is the number of samples correctly classified as positive samples and *FP* (False Positive) is the number of samples wrongly classified as incorrect. In the category of wrong samples, *TN* (True Negative) is the number of samples that are correctly classified as negative samples and *FN* (False Negative) is the number of samples that are wrongly classified as incorrect samples.

## Results and discussion

### Selection of projection dimension *d* in ECA-HBP

**Fig 11:**
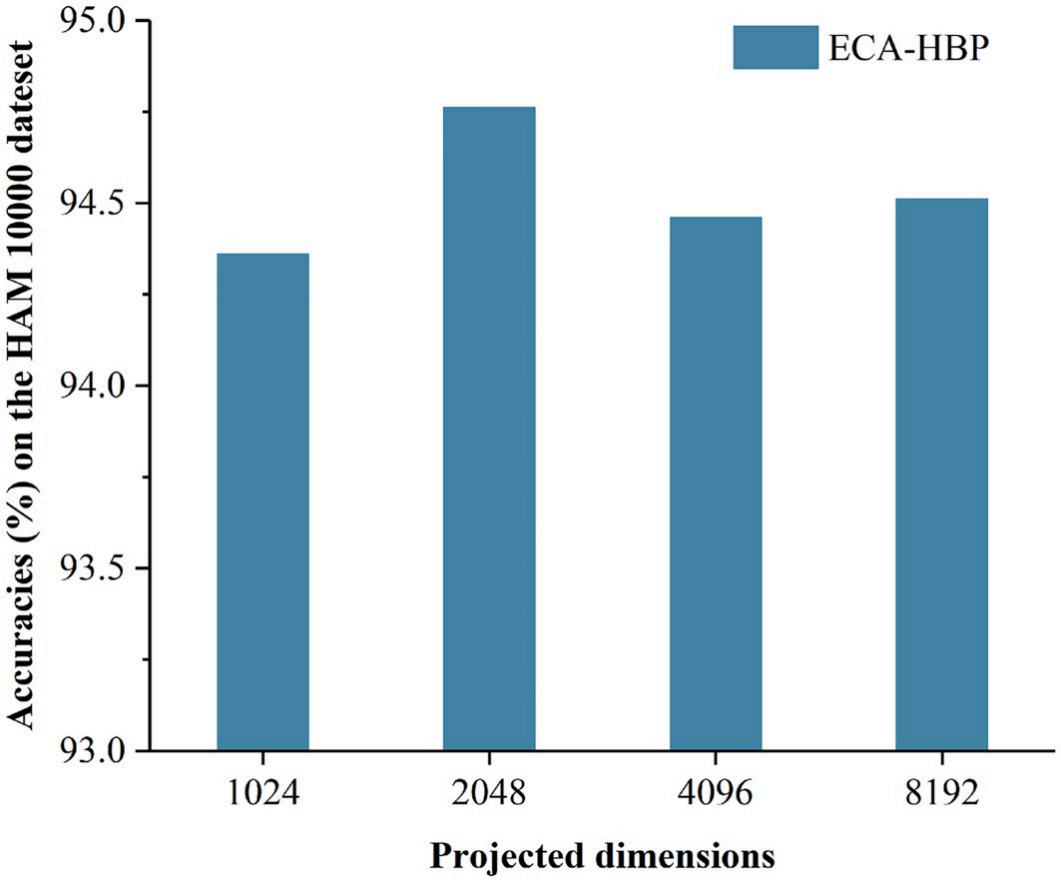
Classification accuracy of different projection dimensions on the HAM10000 dataset.

HBP is to extend the features of different layers in the CNN to high-dimensional space by independent linear mappings, where the projection dimension *d* is defined by the user. To investigate the impact of *d* and to validate the effectiveness of the proposed model, we conduct extensive experiments on the HAM10000 dataset, with results summarized in Fig 11.

It can be seen from Fig 11 that by adjusting the size of the projection dimension *d*, increasing the projection dimension within a certain range can improve the classification performance of the model, but when the projection dimension is too large, the performance of the model begins to decline. When *d* = 2048, the classification accuracy of EFFNet reached the best effect, at this time the classification accuracy rate is 94.76%.

### Results on the HAM10000 dataset

**Fig 12:**
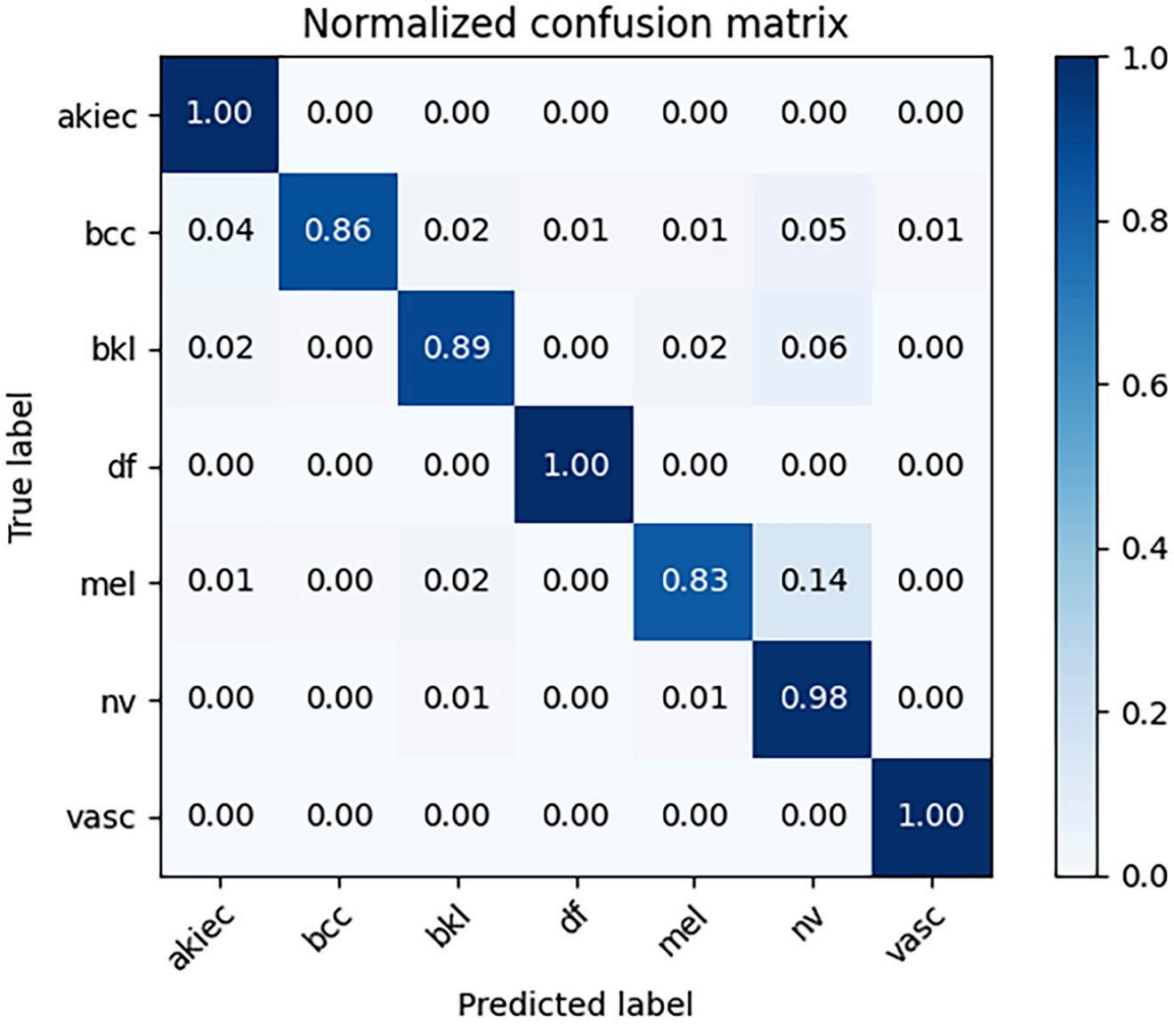
The confusion matrix of skin cancer classification model.

Fig 12 shows the confusion matrix of EFFNet in this paper on the HAM10000 dataset. Through the confusion matrix, we find that EFFNet has a larger error rate and lower accuracy in the mel, bcc and bkl categories, while it performs well in the akiec, df and vasc categories. The evaluation criteria of each category after model classification are shown in Table 2.

**Table 2:** Evaluation indicators for each category of the model.

Type	Acc(%)	Recall(%)	Pre(%)	F1-score(%)
*akiec*	100.00	100.00	85.71	92.31
*bcc*	86.41	86.41	97.80	91.75
*bkl*	89.10	89.10	92.45	90.74
*df*	100.00	100.00	92.00	95.83
*mel*	82.51	82.51	91.09	86.59
*nv*	98.21	98.21	96.27	97.23
*vasc*	100.00	100.00	96.67	98.31

### Ablation experiments

Ablation experiments are often used to explore the rationality of model design, the effectiveness of optimization strategies, or the importance of certain features. The results of ablation experiments of EFFNet on the HAM10000 dataset are shown in Table 3.

**Table 3:** Ablation experiments.

Method	Acc(%)
*EfficientNetV*2 − *M*	83.79
*Pre*_*EfficientNetV*2 − *M*	90.62
*Pre*_*EfficientNetV*2 − *M* + *HBP*	94.56
*Pre*_*EfficientNetV*2 − *M* + *ECA* − *HBP*	94.76
*Proposed*	94.96

As we can see from Table 3, the classification accuracy of the EfficientNetV2-M model on the HAM10000 dataset without transfer learning was only 83.79%, while the model accuracy improved by 6.83% with transfer learning, which proves that transfer learning is suitable for the skin cancer classification task and can improve the accuracy of the model. After adding HBP, the accuracy of the model increased by 3.94%, which indicates that capturing some feature interactions between layers by HBP is effective, while making full use of the extracted features to improve the performance of the model. Adding the ECA mechanism before HBP can reduce the redundant information of feature extraction and retain features that are more important for classification task, thereby increasing the model accuracy by 0.2%. RF is added to the last part of the model for classification, which reduced the overfitting risk of the model and increased the accuracy rate by 0.2%, so as to obtain better classification results.

### Comparison with state-of-the-art architectures

In order to comprehensively evaluate the classification effect of EFFNet, we compared with the convolutional neural networks AlexNet, ResNet50, VGG16, MobileNetV2, EfficientNet-B4 and EfficientNetV2-M on the test sets. Metrics like precision, recall, F1-score, and accuracy are used for performance comparison. The comparison results are shown in Table 4. Compared with other classification models, EFFNet achieved 94.96% accuracy, 93.74% recall, 93.16% precision and 93.24% F1-score on the HAM10000 dataset. The results show that EFFNet proposed in this paper has superior performance and higher accuracy than other models.

**Table 4:** Overall comparison of EFFNet with other state-of-the-art models on the test dataset.

Model	Acc(%)	Recall(%)	Pre(%)	F1-score(%)
*AlexNet*	82.14	81.37	71.99	75.46
*ResNet*50	84.99	82.57	81.29	80.79
*VGG*16	79.80	72.28	67.54	69.02
*MobileNetV*2	83.45	76.99	73.03	75.07
*EfficientNet* − *B*4	84.24	84.19	73.11	72.51
*EfficientNetV*2 − *M*	90.62	90.06	86.66	88.19
*Proposed*	94.96	93.74	93.16	93.24

### Comparison with existing models for skin disease classification

EFFNet was compared with different models that were previously discussed in the related works section and the related observations are reported in Table 5. The metrics that were not reported in the original documents of each work are indicated with a dash(-) in the table. It can be seen from the comparison that the accuracy and recall of EFFNet have improved compared with other models. This is because we not only use feature fusion to capture some feature interactions between layers, but also use ECA mechanism to reduce the interference of redundant information. At the same time, RF is used for final feature classification. The precision of EFFNet is 0.9% lower than that of Xin [23], which may be caused by the different number of training sets after different data enhancement methods, but our accuracy rate is 0.7% higher than that of Xin.

**Table 5:** Overall comparison of EFFNet with existing models on HAM10000 dataset.

Method	Acc(%)	Recall(%)	Pre(%)	F1-score(%)
*Maduranga* [20]	85.0	87.0	87.0	86.0
*Khan* [21]	88.5	88.5	88.7	88.6
*Qian* [22]	91.6	73.5	-	-
*Xin* [23]	94.3	-	94.1	-
*Afza* [24]	93.4	-	93.1	-
*Calderon* [25]	93.2	93.0	92.9	93.2
*Proposed*	95.0	93.7	93.2	93.2

## Conclusion

In this paper, we propose EFFNet based on feature fusion and RF to overcome the disadvantages of unbalanced datasets, redundant information in the extracted features and ignored interactions of partial features among different convolution layers. The experimental results show that the accuracy, recall, precision and F1-score of the model reach 94.96%, 93.74%, 93.16% and 93.24% respectively. Compared with other models, the accuracy rate is improved to some extent and the highest accuracy rate can be increased by about 10%. Although the overall classification accuracy has made some progress, the accuracy of mel, bcc and bkl is lower than other categories, which still needs to be improved. In the future, we will analyze the relationship between dermoscopic images and metadata in clinical data to further explore more information and discover potential patterns to improve the accuracy of classification.
