# Correction: EFFNet: A skin cancer classification model based on feature fusion and random forests

**DOI:** 10.1371/journal.pone.0314412

**Published:** 2024-11-20

**Authors:** Xiaopu Ma, Jiangdan Shan, Fei Ning, Wentao Li, He Li

Fig 10 is uploaded incorrectly. Please see the correct Fig 10 here.

**Fig 10:**
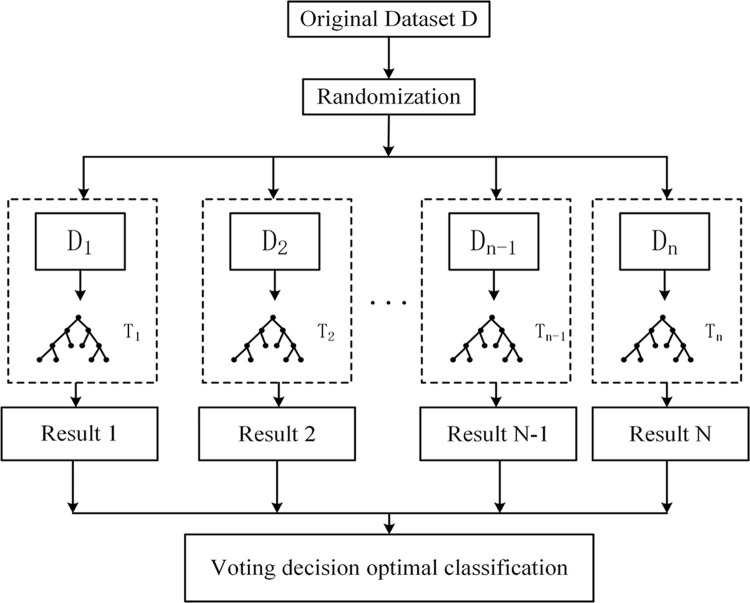
Schematic diagram of random forests algorithm.
